# Retinal Vascular Imaging Markers and Incident Chronic Kidney Disease: A Prospective Cohort Study

**DOI:** 10.1038/s41598-017-09204-2

**Published:** 2017-08-24

**Authors:** Wanfen Yip, Peng Guan Ong, Boon Wee Teo, Carol Yim-lui Cheung, E Shyong Tai, Ching-Yu Cheng, Ecosse Lamoureux, Tien Yin Wong, Charumathi Sabanayagam

**Affiliations:** 1 Singapore Eye Research Institute, Singapore National Eye Centre, Singapore, Singapore; 2 Department of Medicine, Yong Loo Lin School of Medicine, National University of Singapore, Singapore, Singapore; 3 Department of Ophthalmology and Visual Sciences, The Chinese University of Hong Kong, Hong Kong, China; 4 Department of Ophthalmology, Yong Loo Lin School of Medicine, National University of Singapore, Singapore, Singapore; 5 Ophthalmology & Visual Sciences Academic Clinical Program (EYE-ACP), Duke-NUS Medical School, Singapore, Singapore; 6 Centre for Quantitative Medicine, Duke-NUS Medical School, Singapore, Singapore

## Abstract

Retinal microvascular changes indicating microvascular dysfunction have been shown to be associated with chronic kidney disease (CKD) in cross-sectional studies, but findings were mixed in prospective studies. We aimed to evaluate the relationship between retinal microvascular parameters and incident CKD in an Asian population. We examined 1256 Malay adults aged 40–80 years from the Singapore Malay Eye Study, who attended both the baseline (2004–07) and the follow-up (2011–13) examinations and were free of prevalent CKD. We measured quantitative retinal vascular parameters (arteriolar and venular calibre, tortuosity, fractal dimension and branching angle) using a computer-assisted program (Singapore I Vessel Assessment, SIVA) and retinopathy (qualitative parameter) using the modified Airlie house classification system from baseline retinal photographs. Incident CKD was defined as an estimated glomerular filtration rate (eGFR) < 60 mL/min/1.73 m^2^ + 25% decrease in eGFR during follow-up. Over a median follow-up period of 6 years, 78 (6.21%) developed CKD (70.5% had diabetes). In multivariable models, smaller retinal arterioles (hazards ratio [95% confidence interval] = 1.34 [1.00–1.78]), larger retinal venules (2.35 [1.12–5.94] and presence of retinopathy (2.54 [1.48–4.36]) were associated with incident CKD. Our findings suggest that retinal microvascular abnormalities may reflect subclinical renal microvascular abnormalities involved in the development of CKD.

## Introduction

Chronic kidney disease (CKD) is a major public health problem associated with adverse renal and cardiovascular outcomes and premature deaths^1–3^. A better understanding of the underlying pathogenesis associated with CKD is important for further development of screening strategies. Microvascular damage has been shown to play a major role in the onset and development of CKD^4–6^. Experimental studies have reported that renal microvascular alterations including hyalinosis and muscular hyperplasia represent early pathological abnormalities related to CKD^7, 8^. Further, the loss of the renal microvasculature have been hypothesized to correlate directly with the development of glomerular and tubulointerstitial scarring associated with CKD^5^. Despite the importance of renal microvascular changes, the renal microvasculature is not easily examined *in vivo*.

The retinal microvasculature provides an opportunity to study the systemic microvasculature non-invasively. Several studies have reported cross-sectional associations between retinal microvascular changes (i.e. smaller retinal arterioles, presence of retinopathy signs) and CKD^9–12^. However, these associations, specifically between retinal vascular caliber and incident CKD, have not been consistently replicated in prospective studies^13–18^. Previous prospective studies were limited by non-concurrent assessments of retinal imaging and CKD^15^ and high proportion of loss to follow-up or elderly subjects^14, 15^.

Apart from retinal vascular caliber, newer retinal geometry vascular parameters (e.g. retinal fractal dimension and tortuosity) representing the ‘optimality state’ of the retinal microcirculation have been shown to be associated with risk factors of CKD including diabetes and hypertension^19, 20^. Earlier, we reported cross-sectional association between fractal dimension and CKD in two independent studies^10, 21^. Nevertheless, the prospective association between retinal geometry vascular parameters and incident CKD has not been evaluated before.

To address these limitations, we aimed to examine the association of a panel of retinal microvascular imaging markers with incident CKD in a population-based sample of Malay adults aged 40–80 years in Singapore.

## Results

**Figure 1:**
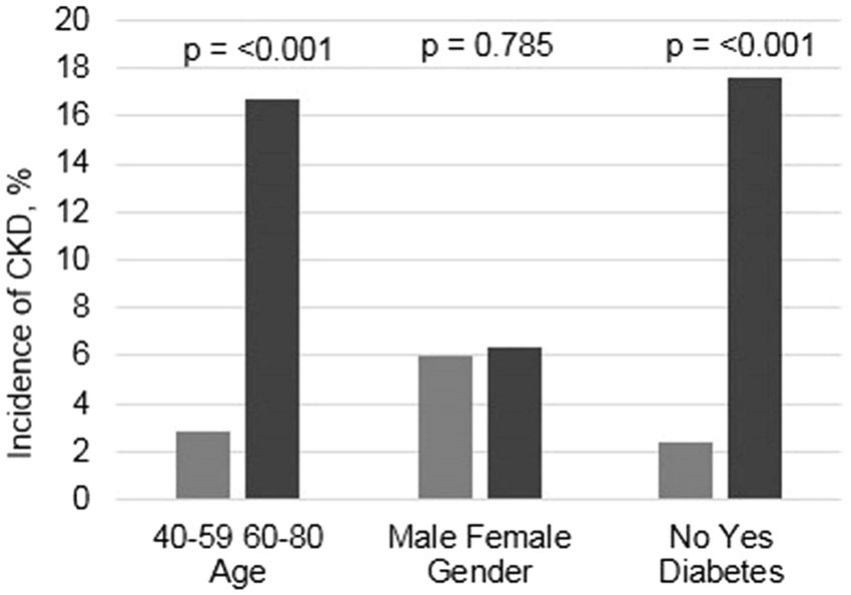
Distribution of incident CKD by subgroups.

Over a median follow-up period of 6 years, 78 participants developed CKD. Of those who developed CKD, 55 (70.5%) had diabetes and 74 (94.9%) had hypertension. Incidence of CKD was significantly higher among those aged 60 years and above and those with diabetes (Fig. 1). A total of 229 (18.2%) participants had a decline in eGFR of>3 mL/min/1.73 m^2^ over the follow-up period.

Table 1 compares the baseline characteristics of participants with and without retinopathy. Participants with retinopathy were more likely to be older, had higher prevalence of hypertension and higher levels of HbA1c, blood glucose and systolic BP. Supplementary Table shows the baseline characteristics of those who were included and excluded from the analysis. Table 2 compares the baseline characteristics of participants stratified by those with and without incident CKD. Participants who developed incident CKD were older, less likely to be secondary/above educated, had higher prevalence of hypertension and diabetes and higher levels of HbA1c, blood glucose, SBP and DBP and lower levels of HDL cholesterol and eGFR compared to those without incident CKD.

**Table 1:** Baseline characteristics of participants with and without retinopathy.

Characteristics	Retinopathy absent	Retinopathy present	*P-value
	(n = 1134)	(n = 122)	
Age, (years)	54.05 (8.77)	55.79 (8.26)	0.037
Gender, males	505 (44.53)	45 (36.89)	0.106
Education, secondary/above	447 (39.42)	41 (33.61)	0.211
Current smoking, yes	223 (19.67)	22 (18.03)	0.666
Alcohol, yes	23 (2.03)	3 (2.46)	0.751
Diabetes, yes	242 (21.34)	71 (58.20)	<0.001
Hypertension, yes	648 (57.14)	85 (69.67)	0.008
HbA1c (%)	6.15 (1.28)	7.68 (2.32)	<0.001
Systolic blood pressure (mm Hg)	140.60 (21.16)	149.41 (23.57)	<0.001
Diastolic blood pressure (mm Hg)	79.21 (10.67)	79.84 (10.72)	0.536
Random blood glucose (mmol/L)	6.12 (2.93)	9.74 (5.72)	<0.001
BMI (kg/m^2^)	26.56 (4.85)	26.98 (4.80)	0.366
Total cholesterol (mmol/L)	5.61 (1.03)	5.54 (1.05)	0.484
HDL cholesterol (mmol/L)	1.37 (0.33)	1.34 (0.33)	0.336
eGFR (mL/min/1.73 m^2^)	83.28 (14.26)	84.02 (14.98)	0.586
hsCRP (mmol/L)	3.59 (7.40)	3.12 (2.84)	0.491
CRAE (µm)	132.70 (11.87)	133.52 (12.33)	0.469
CRVE (µm)	201.90 (16.62)	206.47 (16.65)	0.004
Fractal dimension	1.41 (0.044)	1.42 (0.04)	0.013
Arteriolar tortuosity	0.00030 (0.00013)	0.00030 (0.00014)	0.051
Venular tortuosity	0.00043 (0.00025)	0.00048 (0.00025)	0.385
Arteriolar branching	77.13 (10.47)	78.03 (11.95)	0.376
Venular branching	79.63 (9.93)	79.16 (10.14)	0.620

Abbreviations: BMI: body mass index; CRAE: central retinal artery equivalent; CRVE: central retinal vein equivalent; eGFR: estimated glomerular filtration rate; HbA1c: glycated hemoglobin; HDL: high density lipoprotein; hsCRP: high-sensitivity C-reactive protein. Data presented are mean (standard deviation) or frequency (percentage), where appropriate. *P- value was based on chi-square or t-test where appropriate.

**Table 2:** Baseline characteristics of persons with and without incident CKD at follow-up.

Characteristics	No incident CKD	Incident CKD	*P-value
	(n = 1178)	(n = 78)	
Age (years)	53.67 (8.55)	62.44 (7.30)	<0.001
Gender, males	661 (56.11)	45 (57.69)	0.785
Secondary/above education	472 (40.07)	16 (20.51)	0.001
Current smoking, yes	233 (19.78)	12 (15.38)	0.343
Alcohol, yes	26 (2.21)	0 (0)	0.185
Diabetes, yes	258 (21.90)	55 (70.51)	<0.001
Hypertension, yes	659 (55.94)	74 (94.87)	<0.001
HbA1c (%)	6.20(1.38)	7.77 (2.10)	<0.001
Systolic blood pressure (mm Hg)	140.10 (20.83)	161.90 (22.06)	<0.001
Diastolic blood pressure (mm Hg)	79.07 (10.55)	82.32 (10.99)	0.009
Random blood glucose (mmol/L)	6.24 (3.14)	9.89 (5.77)	<0.001
BMI (kg/m^2^)	26.56 (4.85)	26.98 (4.80)	0.366
Total cholesterol (mmol/L)	5.61 (1.03)	5.55 (1.08)	0.672
HDL cholesterol (mmol/L)	1.37 (0.33)	1.28 (0.32)	0.017
eGFR (mL/min/1.73 m^2^)	83.67 (14.36)	78.69 (12.98)	0.003
hsCRP (mmol/L)	3.56 (7.29)	3.31 (2.77)	0.765
CRAE (µm)	132.77 (11.85)	132.96 (12.85)	0.888
CRVE (µm)	202.21 (16.40)	204.37 (20.36)	0.267
Fractal dimension	1.41 (0.04)	1.40 (0.05)	0.003
Arteriolar tortuosity	0.00031 (0.0001)	0.00026 (0.0001)	0.002
Venular tortuosity	0.00047 (0.0003)	0.00045 (0.0002)	0.495
Arteriolar branching angle	77.21 (10.63)	77.20 (10.51)	0.989
Venular branching angle	79.58 (978)	79.58 (12.28)	0.999
Retinopathy, yes	96 (8.15)	26 (33.33)	<0.001

Abbreviations: BMI: body mass index; CRAE: central retinal artery equivalent; CRVE: central retinal vein equivalent; eGFR: estimated glomerular filtration rate; HbA1c: glycated hemoglobin; HDL: high density lipoprotein; hsCRP: high-sensitivity C-reactive protein. Data presented are mean (standard deviation) or frequency (percentage), where appropriate. *P-value was based on chi-square, or t test, where appropriate.

Table 3 presents the associations between retinal vascular parameters and incident CKD. In tertile analysis, CRAE was not associated with incident CKD in both age, sex adjusted and the multivariable model. In continuous analysis, CRAE showed significant association after adjusting for potential confounders in the multivariable model (hazards ratio [HR] [95% CI] = 1.34 [1.00–1.78]) although the association was not significant in the age, sex-adjusted model. On the other hand, CRVE showed a significant association with incident CKD in both age, sex- adjusted and the multivariable model in tertile analysis but not when analyzed as a continuous variable. Compared to tertile 1 of CRVE, HR (95% CI) of incident CKD was 1.94 (1.11–3.38) in age, sex-adjusted model and 2.35 (1.12–5.94) in the multivariable model. In continuous analysis, although the association of CRVE with CKD was in the positive direction, it did not reach significance. Arteriolar tortuosity showed a positive association in the age, sex adjusted model but lost significance when adjusted for potential confounders in the multivariable model. Presence of retinopathy (HR 2.54, 95% 1.48 to 4.36) at baseline was significantly associated with incident CKD in both age, sex-adjusted and the multivariable models. No significant associations were observed between retinal D_f_, tortuosity and branching angle with incident CKD in either model.

**Table 3:** Association between retinal vascular parameters and incident CKD.

	No at risk	No. with CKD (%)	Age Gender Model		Multivariable Model*	
			HR (95%CI)	P-value	HR (95%CI)	P-value
**CRAE** (µm)						
Tertile 3 (137.24–175.89)	418	26 (6.22)	Referent		Referent	
Tertile 2 (127.42–137.23)	419	20 (4.77)	0.79 (0.45, 1.39)	0.415	0.88 (0.47, 1.67)	0.704
Tertile 1 (90.63–127.41)	419	32 (7.64)	1.00 (0.59, 1.71)	0.979	1.02 (0.51, 2.07)	0.952
CRAE per sd (11.91)↓	1256	78 (6.21)	1.11 (0.90, 1.37)	0.321	1.34 (1.00, 1.78)	0.048
P-trend			0.973		0.932	
**CRVE** (µm)						
Tertile 1 (118.81–195.79)	418	22 (5.26)	Referent		Referent	
Tertile 2 (195.82–208.71)	419	25 (5.97)	1.94 (1.07, 3.49)	0.028	2.65 (1.40, 5.03)	0.003
Tertile 3 (208.72–256.95)	419	31 (7.40)	1.94 (1.11, 3.38)	0.020	2.35 (1.12, 5.94)	0.025
CRVE per sd (16.67)↑	1256	78 (6.21)	1.2 (0.99, 1.46)	0.064	1.25 (0.95, 1.65)	0.116
P-trend			0.021		0.024	
Arteriolar tortuosity, per sd (12.71 × 10^−5^)↓	1256	78 (6.21)	1.35 (1.01, 1.81)	0.045	1.28 (0.96, 1.70)	0.088
Venular tortuosity, per sd (24.63 × 10^−5^)↓	1256	78 (6.21)	1.03 (0.79, 1.35)	0.486	1.24 (0.91, 1.68)	0.171
Fractal dimension, per sd (0.044)↑	1256	78 (6.21)	0.91 (0.73, 1.12)	0.348	0.91 (0.72 1.14)	0.401
Arteriolar branching angle, per sd (10.67)↑	1256	78 (6.21)	1.18 (0.96, 1.45)	0.118	1.14 (0.91, 1.37)	0.306
Venular branching angle, per sd (9.92)↑	1256	78 (6.21)	1.09 (0.87, 1.36)	0.462	1.08 (0.88, 1.32)	0.485
Any retinopathy, yes	122	26 (21.3)	4.12 (2.56, 6.62)	<0.001	2.54 (1.48, 4.36)	0.001

CI: confidence interval; CRAE, central retinal arteriolar equivalent; CRVE, central retinal arteriolar equivalent; HR, hazard ratio; *adjusted for age, gender, baseline glucose levels, baseline SBP, hypertension, smoking status, hsCRP, education level, total cholesterol, HDL cholesterol, baseline eGFR, anti-hypertensive medication and retinal arteriolar caliber/retinal venular caliber (CRAE in models of CRVE and vice versa).

**Figure 2:**
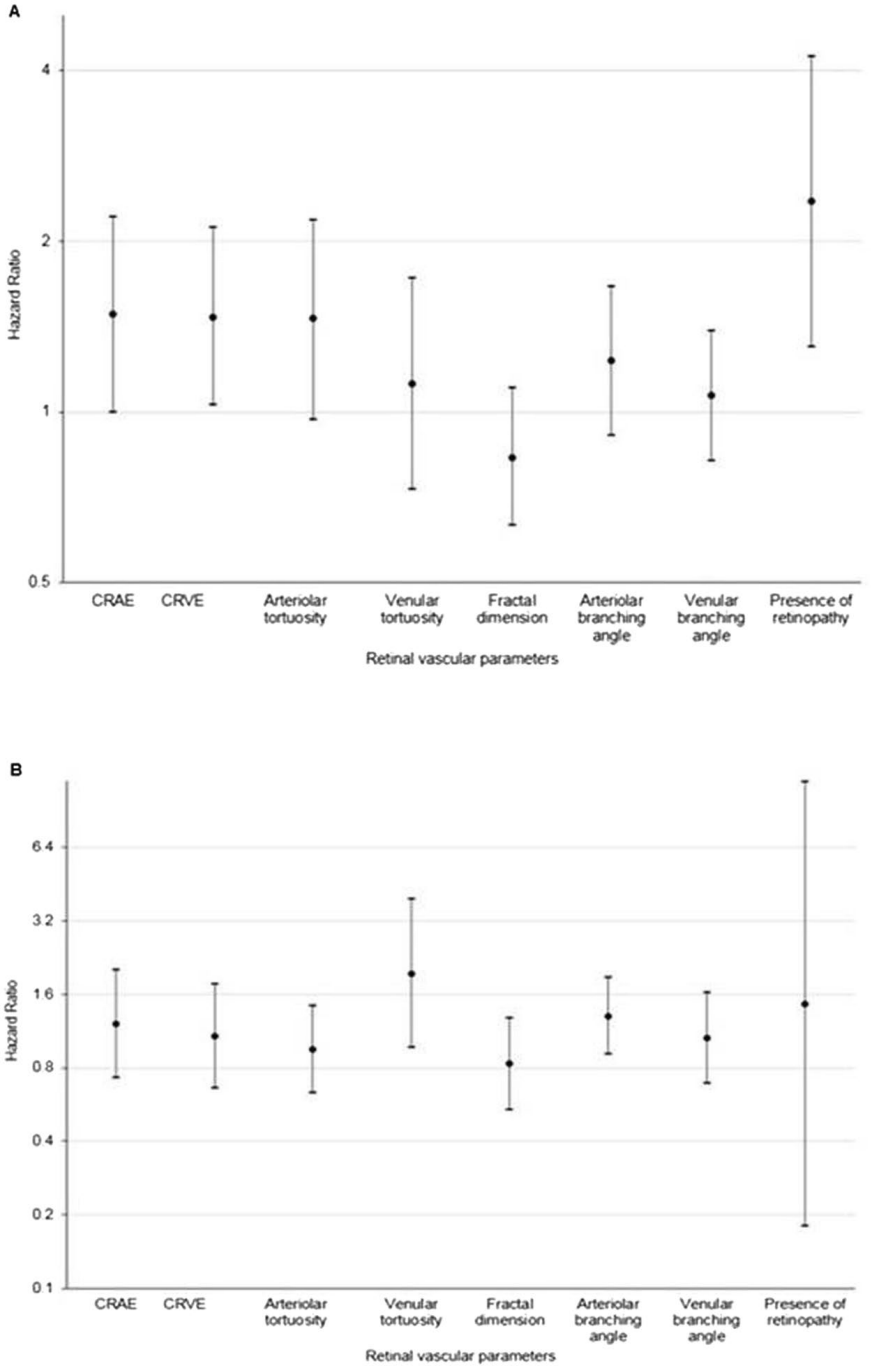
Association between retinal vascular parameters and incident CKD stratified by diabetes status (**A**) presence of diabetes; (**B**) absence of diabetes. CRAE, central retinal arteriolar equivalent; CRVE, central retinal arteriolar equivalent; HR, hazard ratio; CRAE, per sd ↓ (11.74 µm); CRVE per sd ↑ (16.49 µm); Arteriolar tortuosity per sd ↓ (12.4 × 10^−5^); Venular tortuosity, per sd ↓ (21.7 × 10^−5^); Fractal dimension, per sd ↑ (0.043); Arteriolar branching angle, per sd ↑ (10.52); Venular branching angle, per sd ↑ (9.96). Model adjusted for age, gender, baseline glucose levels, baseline SBP, hypertension, smoking status, hsCRP, education level, total cholesterol, HDL cholesterol, baseline eGFR and fellow retinal caliber (CRAE in models of CRVE and vice versa).

Figure 2 shows the association of retinal vascular parameters with incident CKD stratified by diabetes status. Among those with diabetes (Fig 2A), smaller CRAE (per SD decrease: HR 1.49, 95% CI,1.00 to 2.21), wider CRVE (per SD increase: HR 1.47, 95% CI,1.03 to 2.11) and presence of retinopathy (HR 2.35, 95% 1.30 to 4.23) were associated with incident CKD. After additional adjustment for HbA1c and diabetes duration amongst people with diabetes, the association of retinopathy with incident CKD persisted (HR 3.78, 95% 1.72 to 8.32). However, association of CRAE and CRVE with incident CKD lost statistical significance (data not shown). No significant associations were observed between CRAE, or CRVE or retinopathy with incident CKD amongst participants without diabetes (Fig 2B). In analyses stratified by gender, in women, only presence of retinopathy showed significant association with incident CKD (HR 2.83, 95% 1.40 to 5.75). In men, none of the parameters showed any significant associations with CKD (data not shown).

### Supplementary analyses

Incidence of CKD using alternative definition 1 (eGFR 45 + 25% decrease) was 3.1% (n = 39). Repeating the analyses using this definition, associations between retinopathy (HR 3.84, 95% 1.78 to 8.32) and incident CKD remained, but associations between CRAE (HR 1.30, 95% 0.86 to 1.96 per SD decrease) and CRVE (HR 1.22, 95% 0.80 to 1.85 per SD increase) with incident CKD lost statistical significance. Similar to the main analysis, no significant associations were observed between retinal D_f_, tortuosity and branching angle (data not shown). Incidence of CKD using alternative definition 2 (incident CKD and or rapid decline in eGFR) was 24.4% (n = 307). Repeating the analyses using this definition, associations between CRVE (HR 1.17, 95% 1.01 to 1.35), retinopathy (HR 1.54, 95% 1.07 to 2.22) and incident CKD remained significant. No significant associations were observed between CRAE, retinal D_f_, tortuosity and branching angle (data not shown).

## Discussion

In this population-based study, we observed that smaller retinal arterioles, larger retinal venules and presence of retinopathy were associated with an increased risk of CKD over a median follow up of 6 years, independent of potential confounders. These associations between smaller retinal arterioles, larger retinal venules and presence of retinopathy with incident CKD were stronger in participants with diabetes. To our knowledge, the current study is the first one to examine the prospective association of retinal vascular parameters with CKD in an Asian population.

The association between retinopathy and CKD is well established^14, 15^. However, few previous studies have examined the prospective association between retinal vascular parameters and CKD^13–16^. Smaller retinal arterioles were reported to be associated with incident CKD in whites only in the Multi-Ethnic Study of Atherosclerosis (MESA)^13^. In the the Atherosclerosis Risk in Communities Study (ARIC), both retinal arteriolar and venular narrowing were associated with 6-year change in serum creatinine^15^. On the other hand, in the Beaver Dam Chronic Kidney Disease^16^, and in the Cardiovascular Health Study^14^, retinal vascular calibers were not associated with CKD. It is possible that selective mortality and survival bias may have masked the associations between retinal vascular caliber and renal impairment. Further, previous studies^14, 15^ did not assess exposure (retinal imaging) and outcome (CKD) measurements at baseline concurrently. For e.g. in ARIC study, authors compared retinal data collected at the third examination with the change in serum creatinine levels and development of renal dysfunction between the second and the fourth examinations^15^.

In a previous study evaluating the association of retinal microvascular abnormalities with incident ESRD, we observed significant association of retinopathy, but not other retinal parameters with ESRD^18^. The lack of association of retinal vascular parameters could be due to the smaller number of ESRD events (n = 33) or selective mortality of those with smaller retinal arterioles and ESRD^18^. In a recent study by Grunwald *et al*.^17^, retinal vascular calibre was not significantly associated with incident ESRD in a cohort of patients with CKD. In the current study, in persons with diabetes, smaller retinal arterioles, larger retinal venules and retinopathy were significantly associated with incident CKD. Similar to our findings, the Wisconsin Epidemiologic Study of Diabetic Retinopathy (WESDR), reported that retinal venular dilation was associated with gross proteinuria and renal insufficiency in persons with type 1 diabetes^22^.

Retinal arteriolar narrowing has been hypothesized to represent a dysregulation of the renin-angiotensin and endothelin^23, 24^. Importantly, over expression of endothelin, a potent vasoconstrictor secreted by vascular endothelial cells, has been hypothesized to be associated with sclerotic renal changes and progression of kidney disease^25^. Furthermore, experimental data from kidney biopsies of persons with type 1 diabetes have shown that narrower retinal arteriolar caliber is morphologically related to extracellular matrix accumulation^26^, which ultimately leads to a decrease in eGFR^27^. Together, it plausible that these processes may provide a common pathophysiologic link between retinal arteriolar narrowing and decrease in eGFR. The pathological processes underlying the association between retinal venular widening and incident CKD are not clear. Larger venular diameters and increased blood flow has been reported to be associated with diabetic status^28–33^. In addition, retinal venular widening and DR have been postulated to be a result of endothelial damage and inflammatory processes^34^. Clinically, retinal venular widening and DR represent thickening of basement membrane, and increased leakage, which is also observed in CKD^35^. Therefore, it is possible that retinal venular widening and DR are reflective of cumulative renal microvascular damage which eventually results in CKD.

The strengths of our study include its population-based sample, quantitative and masked evaluation of retinal vascular parameters, standardized measurement of renal function, and the availability of information on potential confounding factors. Our study has some limitations. First, we could not adjust for albuminuria in the multivariable model since data was not available in most of the participants. Second, since majority of the participants had hypertension (58.4%), we could not stratify the population by hypertension status. Third, antihypertensive medication use was not assessed in detail (e.g. ACE inhibitors). Fourth, since majority of the participants who developed CKD belonged to stage 3 (63 of 78 incident CKD, i.e. 81%), we were unable to evaluate if the addition of retinal vascular parameters aid in risk-stratification of CKD.

In conclusion, a population-based sample of Malay adults, we found that the presence of retinal microvascular changes including smaller retinal arterioles, larger retinal venules and presence of retinopathy were associated with increased risk of CKD. Our findings suggest that retinal microvascular abnormalities may reflect early subclinical damage in the renal microvasculature that is subsequently associated with development of CKD.

## Methods

### Study Population

We utilized data from the Singapore Malay Eye Study (SiMES), a population-based cohort study. In brief, 3280 adults aged between 40 to 80 years recruited from the community using an age-stratified random sampling method attended the baseline examination in 2004 to 2006 (78.7% response rate). The methodology and objectives of the study population have been reported in detail elsewhere^36, 37^. Of the 2636 eligible participants, 1901 returned for the follow-up examination in 2004–06 (response rate: 72.1%). Written, informed consent was obtained from each participant; the study conducted adhered to the Declaration of Helsinki. Ethical approval was obtained by the Singapore Eye Research Institute Institutional Review Board. After excluding participants who had missing data on estimated glomerular filtration rate (eGFR) levels (n = 101), prevalent CKD (n = 307), ungradable baseline retinal photographs (n = 111), missing data on covariates (n = 76) and history of cardiovascular disease (CVD; n = 50), 1256 participants were included for the current analysis.

### Measurement of Retinal Vascular Parameters

Retinal fundus photographs of both eyes were taken after dilating the pupils with 1% tropicamide and 2.5% phenylephrine hydrochloride, using a digital non-mydriatic retinal camera (CR-DGi with a 10D SLR backing; Canon, Tokyo, Japan). We used a semi-automated computer-assisted program (Singapore I Vessel Assessment [SIVA], version 1.0) to measure retinal microvascular parameters quantitatively from digital retinal photographs. Trained graders, masked to participant characteristics, executed the SIVA program to measure the retinal microvasculature.

SIVA automatically identifies the optic disc and places a grid with reference to the center of optic disc. Retinal arterioles and venules were also automatically identified. All visible vessels coursing through the specified zone (0.5 disc diameter – 2.0 disc diameter) were measured. Graders were responsible for the visual evaluation of SIVA-automated measurements and manual intervention if necessary, following a standardized grading protocol. The intra- and inter-grader reliability was assessed and reported previously^38^. Retinal vascular parameters (arteriolar and venular caliber, fractal dimension, tortuosity and branching angle) were automatically measured and quantified by the SIVA program^38, 39^. Based on the revised Knudtson-Parr-Hubbard formula, the retinal arteriolar and venular calibers were summarized as central retinal artery equivalent (CRAE) and central retinal vein equivalent (CRVE) respectively^40^. CRAE represents the average width of retinal arterioles while CRVE represents the average width of retinal venules.

Retinal vascular fractal dimension (D_f_) represents a ‘global’ measure that summarizes the entire branching pattern of the retinal vascular tree. Retinal D_f_ was calculated from a skeletonized line tracing using the box counting method; these represent a “global” measure that summarizes the whole branching pattern of the retinal vascular tree^41^. Larger values indicate a more complex branching pattern. Retinal vascular tortuosity was derived from the integral of the curvature square of along the path of the vessel, normalized by the total path length, taking into account the bowing and points of inflection^20, 42^. Estimates were summarized as retinal arteriolar tortuosity and retinal venular tortuosity. Smaller values indicate straighter vessels. Retinal vascular branching angle was defined as the first angle subtended between two daughter vessels at each vascular bifurcation^38, 43^. The estimates were summarized as retinal arteriolar branching angle and retinal venular branching angle, representing the average branching angle of arterioles and venules of the eye, respectively. Larger values indicate that the widths of the two daughter branching vessels are more symmetric^38^.

### Retinopathy Signs

Retinopathy was considered present if any characteristic lesion (microaneurysms, haemorrhages, cotton wool spots, intraretinal microvascular abnormalities, hard exudates, venous beading and new vessels) was present^44^. For each eye, a retinopathy severity score was assigned accordingly and retinopathy was defined as being present if the retinopathy score (a scale modified from the Airlie House classification system) was at level 15 or higher^45^.

### Ascertainment of Incident CKD

Glomerular filtration rate was estimated from serum creatinine using the Chronic Kidney Disease Epidemiology Collaboration (CKD-EPI) equation^46^. Serum creatinine was measured using an enzymatic method calibrated to the National Institute of Standards and Technology (NIST) Liquid Chromatography Isotope Dilution Mass Spectrometry (LC-IDMS) method recommended by the National Kidney Disease Education Program and traceable to NIST SRM967^47^.

Incident CKD was defined as eGFR < 60 mL/min/1.73 m^2^ accompanied by a decrease in eGFR of at least 25% over the follow-up period^48^ among subjects free of CKD at baseline. For sensitivity analyses, we used two alternate definitions of incident CKD: 1) eGFR < 45 mL/min/1.73 m^2^ accompanied by a decrease in eGFR of at least 25% over the follow-up period among subjects free of CKD at baseline^48^ 2) composite of incident CKD (our main definition) and/or rapid decline in eGFR. Rapid decline in eGFR was defined as annualized eGFR rate of >3 mL/min/1.73 m^2^/year where annualized eGFR rate is calculated as the difference in eGFR between the baseline and follow-up visit divided by elapsed time in years^49^.

### Assessment of covariates

Information on participants’ demographic characteristics, lifestyle (current smoking), personal and medical history was obtained by using a standardized questionnaire administered by trained personnel. Education was categorized into primary/below education or secondary/above education. CVD was defined as self-reported history of stroke, myocardial infarction or angina. Age was defined as the age at the time of baseline examination. Height was measured in centimeters using a wall-mounted measuring tape and weight was measured in kilograms using a digital scale. Body mass index (BMI) was calculated as body weight (in kilograms) divided by body height (in meters squared). Systolic blood pressure (SBP) and diastolic blood pressure (DBP) were measured using a digital automatic BP monitor (Dinamap model Pro Series DP110X-RW, 100V2, GE Medical Systems Information Technologies Inc, Milwaukee, WI), after the subject was seated for at least 5 minutes. BP was measured twice at intervals of 5 minutes apart in a seated position. If these measurement differed by >10 mm Hg of SBP, or >5 mm Hg of DBP, then a third measurement was made. The mean between the 2 closest readings was then taken as the BP of that individual. Hypertension was defined as systolic BP of ≥140 mm Hg, diastolic BP of ≥90 mm Hg, or self-reported previously diagnosed hypertension. Antihypertensive medication use was defined as self-reported use of antihypertensive medications.

Non-fasting venous blood samples were analyzed at the National University Hospital Reference Laboratory for measuremnt of plasma glucose, serum total cholesterol, glucosylated hemoglobin (HbA1c) high-density lipoprotein (HDL) cholesterol, low-density lipoprotein cholesterol, and high-sensitivity C-reactive protein (hsCRP). Diabetes mellitus was defined as a casual plasma glucose measurement of ≥200 mg/dL (11.1 mmol/L), self-reported physician-diagnosed diabetes, use of glucose-lowering medication, or HbA1c ≥6.5%^50^.

### Statistical Analysis

All statistical analyses were performed using STATA statistical software (Version 10, StataCorp, College Station, Texas). The outcome of interest for our study was incident CKD. The main exposures of interest were CRAE, CRVE, retinal vascular tortuosity, retinal D_f_, retinal vascular branching angle and presence of retinopathy. Each parameter was analysed separately with outcome CKD. Retinal vascular calibers (CRAE and CRVE) were analysed in tertiles as well as continuous variables (per each standard deviation increase/decrease). As previous studies have shown smaller retinal arteriole (CRAE)^13^ and larger retinal venule (CRVE)^22^ to be associated with CKD. In tertile analyses, tertile 3 (wider retinal arteriole) was used as the reference for CRAE, and tertile 1 (smaller retinal venule) was used as the reference for CRVE. We compared baseline characteristics between participants 1) with and without retinopathy and 2) with and without incident CKD at follow-up, by employing the chi-squared test or independent t-test as appropriate. Cox proportional-hazards regression was performed to examine the relation between retinal vascular parameters (CRAE, CRVE, retinal tortuosity, retinal D_f_ and retinal branching angle, retinopathy) and CKD, in two models: model 1 adjusting for age and sex; model 2 additionally adjusting for education level, baseline eGFR, glucose levels, SBP, hypertension, smoking status, anti-hypertensive medications, hsCRP, total cholesterol, HDL cholesterol and fellow retinal vessel caliber (e.g. CRAE in models including CRVE and vice versa). P-trend was examined using CRAE and CRVE categories as continuous variables in the corresponding multivariable models. To examine the consistency of the association, we performed subgroup analyses stratified by sex and diabetes status using retinal vascular parameters as continuous variables. To test the robustness of association, we repeated the main analyses using alternative definitions of incident CKD.

### Availability of data and material

As the study involves human participants, the data cannot be made freely available in the manuscript, the supplemental files, or a public repository due to ethical restrictions. Nevertheless, the data are available from the Singapore Eye Research Institutional Ethics Committee for researchers who meet the criteria for access to confidential data.

## Electronic supplementary material

Supplementary Information
