# Supplementary material for: Retinal Vascular Imaging Markers and Incident Chronic Kidney Disease: A Prospective Cohort Study
Source: Sci Rep. 2017 Aug 24;7:9374. doi: 10.1038/s41598-017-09204-2 (PMC5570935; doi:10.1038/s41598-017-09204-2)
Supplement: Supplementary file 1 — Supplementary Information [file 41598_2017_9204_MOESM1_ESM.pdf]

Retinal Vascular Imaging Markers and Incident Chronic Kidney Disease: A Prospective Cohort Study

Wanfen Yip, Ong Peng Guan, Boon Wee Teo, Carol Yim-lui Cheung, E Shyong Tai, Ching-Yu Cheng, Ecosse Lamoureux, Tien Yin Wong, Charumathi Sabanayagam

Supplementary Table S1. Baseline characteristics comparing between participants who were included to those who were excluded from the study

Characteristics	Included (n=1256)	Excluded (n=645)	*P-value
Age (years)	54.21 (8.73)	62.20 (10.37)	**<0.001**
Gender, males	550 (43.79)	314 (48.68)	**0.043**
Education, secondary/above education	488 (38.85)	149 (23.24)	**<0.001**
Current smoking, yes	245 (19.51)	101 (15.73)	**0.044**
Alcohol, yes	26 (2.07)	6 (0.094)	0.070
Diabetes, yes	313 (24.92)	241 (41.77)	**<0.001**
Hypertension, yes	733 (58.36)	494 (77.92)	**<0.001**
HbA1c (%)	6.30 (1.48)	6.53 (1.52)	**0.002**
Systolic blood pressure (mm Hg)	141.45 (21.55)	149.75 (23.91)	**<0.001**
Diastolic blood pressure (mm Hg)	79.27 (10.67)	79.75 (11.71)	0.367

Random blood glucose (mmol/L)	6.47 (3.47)	7.00 (3.79)	**0.004**
BMI (kg/m^2)	26.60 (4.84)	26.57 (4.79)	0.872
Total cholesterol (mmol/L)	5.60 (1.03)	5.64 (1.24)	0.526
HDL cholesterol (mmol/L)	1.37 (0.33)	1.34 (0.33)	0.070
eGFR (mL/min/1.73 m^2)	83.35 (14.32)	63.24 (20.08)	**<0.001**
hsCRP (mmol/L)	3.54 (7.09)	4.00 (6.97)	0.196
CRAE (µm)	132.78 (11.91)	134.53 (12.87)	**0.008**
CRVE (µm)	202.34 (16.67)	203.21 (17.96)	0.344
Fractal dimension	1.41 (0.04)	1.40 (0.05)	**0.0002**
Arteriolar tortuosity	0.00030 (0.00013)	0.00029 (0.00014)	0.071
Venular tortuosity	0.00047 (0.00024)	0.00046 (0.00020)	0.487
Arteriolar branching	77.21 (10.62)	76.50 (11.68)	0.224
Venular branching	79.58 (9.95)	79.59 (10.88)	0.984

Abbreviations: BMI: body mass index; CRAE: central retinal artery equivalent; CRVE: central retinal vein equivalent; eGFR: estimated glomerular filtration rate; HbA1c: glycated hemoglobin; HDL: high density lipoprotein; hsCRP: high-sensitivity C-reactive protein.

Data presented are mean (standard deviation) or frequency (percentage), where appropriate.

*P- value was based on chi-square or t-test where appropriate.
